# Global Profiling of Post-Translationally Modified Crustacean Neuropeptidome Trends Affiliated with Feeding Activity

**DOI:** 10.1021/jasms.6c00017

**Published:** 2026-03-18

**Authors:** Lauren Fields, Kendra G. Selby, Meghan M. Hayes, Paul Corsetti, Tong Gao, Lingjun Li

**Affiliations:** † Department of Chemistry, 5228 University of Wisconsin-Madison, 1101 University Avenue, Madison, Wisconsin 53706, United States; ‡ Department of Chemical and Biological Engineering, University of Wisconsin−Madison, 1415 Engineering Drive, Madison, Wisconsin 53706, United States; § Department of Molecular & Environmental Toxicology, 1300 University Ave #6152, Madison, Wisconsin 53706, United States; ∥ School of Pharmacy, University of Wisconsin-Madison, 777 Highland Avenue, Madison, Wisconsin 53705, United States; ⊥ Lachman Institute for Pharmaceutical Development, School of Pharmacy, University of Wisconsin-Madison, Madison, Wisconsin 53705, United States; # Wisconsin Center for NanoBioSystems, School of Pharmacy, University of Wisconsin-Madison, Madison, Wisconsin 53705, United States

**Keywords:** neuropeptide, post-translational modifications, feeding, crustacean, amidation, acetylation, methylation, peptidomics, mass spectrometry, neuromodulation

## Abstract

Neuropeptides modulate a diverse range of physiological functions, including those associated with feeding. Post-translational modifications (PTMs) contribute significantly to the dynamic nature of neuropeptide isoforms, influencing their functional diversity. Mass spectrometry is the gold-standard analytical technique for peptidomic analyses and is complemented by computational methods for peptide identification; however, the computational search space becomes increasingly difficult to manage as more potential modifications are considered. Using innovative approaches capable of addressing the vast combinations of possible modifications, such as the PEAKS PTM search algorithm, we globally profiled the neuropeptidome of*Cancer borealis*­(Jonah crab) to investigate the role of PTMs in feeding- and appetite-related processes over time. Through an in-depth examination of several notable modifications, we proposed PTM-associated motifs for neuropeptides, which may enhance future identification capabilities. Furthermore, this work revealed neuropeptides that were characteristically modified depending on the crab’s feeding status and time post-feeding, suggesting potential biological significance. This study represents the first large-scale investigation of the modified crustacean neuropeptidome, providing new insights into the regulatory implications of PTMs in biological systems.

## Graphical abstract

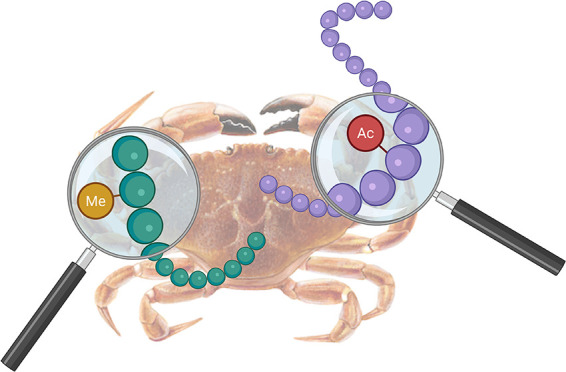

## Introduction

Computationally, investigating the vast number of possible post-translational modifications (PTMs) and their combinations poses significant challenges due to the rapid expansion of search space, presenting a limitation when performing discovery-based mass spectrometry analyses. An alternative to these “closed searches”, which rely on predefined search parameters, is the use of “open searches”. Open searches allow for a wide precursor-mass error tolerance, enabling peptide identifications that can later be mapped to specific modifications.^1,2^ This strategy has dramatically accelerated the discovery of novel modifications.^3^ Although a few studies have focused on specific PTMs,^4^ a comprehensive, large-scale PTM analysis using this approach has not yet been conducted. Leveraging open-search strategies, researchers have profiled PTM contributions to complex human conditions, such as glioblastoma and gastric cancer,^5^ and have explored under-studied sample types including the microbiome,^6^ glycoproteomes,^6^ and chemical transformations induced by sample preparation.^7^

Recent studies have highlighted that more “niche” PTMs play dynamic and important roles in regulatory mechanisms, cellular functions, and homeostasis. For instance, lactylation has gained attention for its role in metabolism.^8^ Considering that the majority of mass spectrometry scans remain unassigned, it is reasonable to assume that some of these may result from overlooked PTMs.^9,10^ Neuropeptides represent one of the most diverse types of signaling molecules, which regulate key biological functions and are extensively modified. The field of neuropeptidomics has benefited substantially from innovations in mass spectrometry and downstream data analysis. Thus, open-modification searching presents an opportunity to further discovery efforts and determine yet-uncharacterized modifications at play in neuropeptide signaling, specifically in response to feeding.

Feeding is a complex biological process whose underlying mechanisms remain elusive. One contributing factor is the flexibility of neuromodulation, which is greatly influenced by hormonal activity.^11,12^ In the context of feeding, hormonal responses have been linked to complex phenomena such as perceived food value,^11^ olfactory behavior,^13^ adjustments in sweetness perception,^12^ hunger signaling,^14^ and appetite control.^15^ Additionally, several of these systems, when examined in depth, are modulated by multiple neural circuits.^13,15^ At a deeper molecular level, many of these hormones, including the well-known insulin hormone, encode neuropeptides, dynamic signaling molecules that govern these downstream functions.^13^

To disentangle these highly convoluted neural signaling profiles, simpler model organisms have proven invaluable for revealing comodulatory processes in ways that remain highly relevant to higher-order organisms. For example, neuropeptide Y is among the most widely studied neuropeptides and is analogous to the RYamide family found in invertebrates.^16^ The Jonah crab (*Cancer borealis*) is a commonly employed model organism in this realm and has been extensively studied for its gastric mill and pyloric rhythm neural circuits, which influence chewing and filtering of chewed food, respectively.^17^

The *in vivo* actions of neuropeptides are mediated through interactions with their cognate G-protein coupled receptors (GPCRs).^18,19^ Moreover, PTMs are known to modulate neuropeptide function, and in many cases are required for full biological activity.^20–23^ For example, a key component in neuropeptide signaling that has been largely overlooked is PTMs. C-terminal amidation and N-terminal cyclization of glutamine or glutamic acid to pyroglutamic acid (pyro-Glu) minimize *in vivo* degradation by carboxypeptidases and aminopeptidases, respectively.^18,19^ In mass spectrometry (MS)-based neuropeptidomics, neuropeptides are often classified as “protein-like” or ”nonprotein-like”, referencing terminology from the more mature field of proteomics. Interestingly, C-terminal amidation or pyroglutamic acid formation are considered artifactual in proteomics, but are biologically relevant in neuropeptidomics.^18^ Consequently, the extensive knowledge of PTMs in proteomics does not directly translate to neuropeptidomics, necessitating dedicated analysis. Furthermore, the limited understanding of neuropeptide-PTM relationships likely extends much deeper and may illuminate fundamental aspects of neural signaling.

Recognizing the temporal nature of feeding, which spans from ingestion to complete clearance over approximately 24 h, we aimed to profile neuropeptide expression throughout this cascade.^24^ Beyond the neuropeptide backbones, we specifically examined the frequency and distribution of PTMs across the feeding timeline. Furthermore, we assessed the biochemical conditions associated with these modifications by defining conserved sequence regions, ultimately yielding the first PTM-associated motifs for neuropeptides.

## Materials and Methods

### Chemicals and Materials

Unless otherwise denoted, all reagents were Optima grade. Formic acid was purchased from Sigma-Aldrich (St. Louis, MO). Methanol (MeOH), water (H_2_O), acetic acid (AA), sodium chloride (NaCl), potassium chloride (KCl), calcium chloride (CaCl_2_), magnesium chloride (MgCl_2_), sodium hydroxide (NaOH), acetonitrile (ACN), and any other chemicals or solvents were purchased from Fisher Scientific (Pittsburgh, PA). Applicable solutions to this work include acidified MeOH, composed of 90 MeOH/9 H_2_O/1 AA (v/v/v), and crustacean physiological saline. Saline was produced with 440 mM NaCl, 11 mM KCl, 13 mM CaCl_2_, 26 mM MgCl_2_, 11 mM Trizma base, and 5 mM maleic acid, and subsequently adjusted to pH 7.45 with NaOH.

### Animals and Feeding Experiments

Male Jonah crabs,*C. borealis*, were acquired from Global Market (Madison, WI). Prior to tissue acquisition, crabs were equilibrated for 2 weeks. Aquarium conditions included an artificial seawater environment (35 parts per thousand salinity) at 12–13 °C and alternating 12 h light/dark cycle. For feeding analysis, crabs were fed 4 g thawed tilapia, observed until feeding was complete, and allowed to rest for the targeted amount of time. Selected time points included 0 h, 30 min, 1 h, and 24 h post-feeding. For each fed crab, an unfed (control) crab was also dissected. For dissection, crabs were placed in packed ice for 30 min to achieve anesthetization. From each crab, the pericardial organs (POs), sinus glands (SGs), thoracic ganglion (TG), brain, and stomatogastric nervous system (STNS) were collected. Raw tissues were submerged in acidified MeOH and stored at −80 °C until extraction.

### Extraction and Desalting

Sample preparation of neuropeptides for MS analysis was conducted as described previously.^25^ Briefly, preserved samples were homogenized via hand-held ultrasonic homogenizer at an amplitude of 60%, and subsequently centrifuged for 1 h at 16k RCF, retaining the supernatant. Three bioreplicates of each sample were pooled and desalted via ZipTips (Agilent) according to manufacturer instructions, eluting in a gradient of 25% ACN, 50% ACN, and 75% ACN in H_2_O.

### Mass Spectrometry Data Acquisition

LC-MS/MS was applied for untargeted analysis of neuropeptides extracted from *C. borealis* tissue. LC separation took place via a homemade 15 cm (75 μm internal diameter) capillary column packed with ethylene bridged hybrid 1.7 μm C18 beads. The integrated emitter tip was aligned with the MS inlet of the Thermo Q-Exactive mass spectrometer connected to a Waters nanoACQUITY UPLC system. Mobile phases A and B for chromatographic separation were 0.1% FA in H_2_O and acetonitrile, respectively. Gradient elution was conducted as follows in terms of mobile phase B at 300 nL/min: 0 min, 0%; 18 min, 0%; 18.5 min, 10%; 88 min, 20%; 128 min, 35%; 138 min, 75%; 139 min, 75%; 140 min, 95%; 150 min, 95%; 151 min, 0%; and 156 min, 0%. Full MS^1^ scans were acquired from 300 to 2000 *m*/*z* at a resolution of 70,000; automatic gain control (AGC) target, 1 × 10^6^; maximum injection time, 250 ms. The top 20 most abundant precursor ions were selected for fragmentation by higher-energy collision dissociation (HCD) for data-dependent acquisition (DDA). Other DDA MS^2^ parameters include dynamic exclusion window, 40.0 s; isolation window, 2.0 *m*/*z*; fixed first mass, 120.0 *m*/*z*; normalized collision energy (NCE), 30; resolution, 17,500; AGC target, 2 × 10^5^; and maximum injection time, 250 ms. Data were acquired for one technical replicate of each sample.

### Data Analysis

Raw MS data were searched against an in-house developed crustacean neuropeptide database containing 891 unique neuropeptides^26^ using PEAKS Studio version 10.6 (Bioinformatics Solutions Inc.). General search parameters for both the closed and open search were parent mass tolerance, 20.0 ppm; fragment mass error tolerance, 0.02 Da; precursor mass search type, monoisotopic; enzyme, none; maximum missed cleavages, 100; digest mode, no digestion; and maximum variable PTM per peptide, 3. Variable modifications for the closed search included C-terminal amidation, oxidation of M, and cyclization of N-termini glutamine or glutamic acid to pyroglutamic acid (pyro-Glu). The open search considered these four as well as 308 additional variable modifications, with at most 2 variable PTMs per peptide, and a *de novo* score threshold of 15%. Search results were exported with confidence filters applied at the peptide and protein levels. Identified results were filtered at the peptide level to 1% false discovery rate (FDR) in the closed search, and the corresponding PEAKS score (−10 lg P) was used for filtering the open search of the same sample. Protein level filters included PEAKS Score >0, at least 1 unique peptide, and with significant peptides. Peptide motifs were evaluated by aligning all peptides containing a particular PTM and conducting WebLogo analysis (https://weblogo.berkeley.edu/logo.cgi).

## Results and Discussion

Identifying post-translational modifications implicated in neuropeptide signaling is paramount to understanding the underpinnings of complex biological processes such as feeding. To achieve this, we applied a broad-spectrum approach to survey putative post-translationally modified peptides. One of the most efficient strategies for this purpose is the use of open-modification database-searching strategies.

### Application of Open-Modification Searches to Crustacean Neuropeptides

Given the well-established importance of PTMs in biological processes, it is no surprise that since the advent of database searching, researchers have sought new approaches to extract modified peptides from mass spectral data.^27–30^ A variety of methods have been proposed, including two-stage algorithms,^31^ sequence alignment strategies,^32^ spectral library approaches,^33^ ion indexing,^34^ and error-tolerant methods,^35,36^ such as the one evaluated in this work. More recently, the integration of several approaches has enhanced confidence in identifications while enabling the discovery of novel peptides, often through the incorporation of *de novo* sequencing into these workflows.^2,37^ *De novo* sequencing has experienced periodic surges and declines in popularity, historically remaining underutilized due to computational demands that were once prohibitive.^38^ PEAKS is widely used software for analyzing challenging, low-abundance samples, including neuropeptides. By integrating *de novo* sequencing with database searching, it enhances sensitivity for detecting complex analytes. Consequently, its utility for identifying potentially modified peptides was of particular interest.^39^ To investigate the PTM landscape of neuropeptides in this study, PEAKS PTM was employed in parallel with the traditional PEAKS DB searching pipeline.

Our first objective was to determine whether peptide identities were consistent between the two search strategies. For example, a rarely observed neuropeptide might be identified within the larger search space offered by open-modification searching, which allows unrestricted modification assessment. To this end, we examined what we refer to as “peptide backbones”, defined as the amino acid sequence regardless of any modifications. In contrast, we also assessed “peptide identifications”, which include both the backbone and any detected modifications.

Because PTM expression is expected to vary across feeding and digestion, we evaluated all predefined time points using both search strategies. The open and closed search methods yielded similar numbers of backbone identifications across all tissues, time points, and feeding statuses (Figures 1A, S1A). However, there was a substantial increase in the number of unique peptide identifications with the open search strategy (Figures 1B, S1B). Further inspection revealed that the open search identified several peptides bearing hallmark features of active neuropeptides, namely N-terminal pyro-glutamate (pyro-Glu) modification formed by cyclization of either glutamine or glutamic acid, as well as C-terminal amidation.^16^ These common neuropeptide modifications frequently co-occurred with previously unannotated PTMs, contributing to the overall increase in identifications. Importantly, despite this expanded search space, there was no significant difference in the distribution of the corresponding PEAKS scores (i.e., −10 log P) between closed and open searches, indicating that search rigor was maintained in both scenarios (Figure 1C). It is also worth noting that the PEAKS score reflects a metric similar to the widely used false-discovery rate (FDR), but this specialized scoring approach has been shown to yield more reproducible results in data sets with only a few hundred expected identifications.^40^

**Figure 1:**
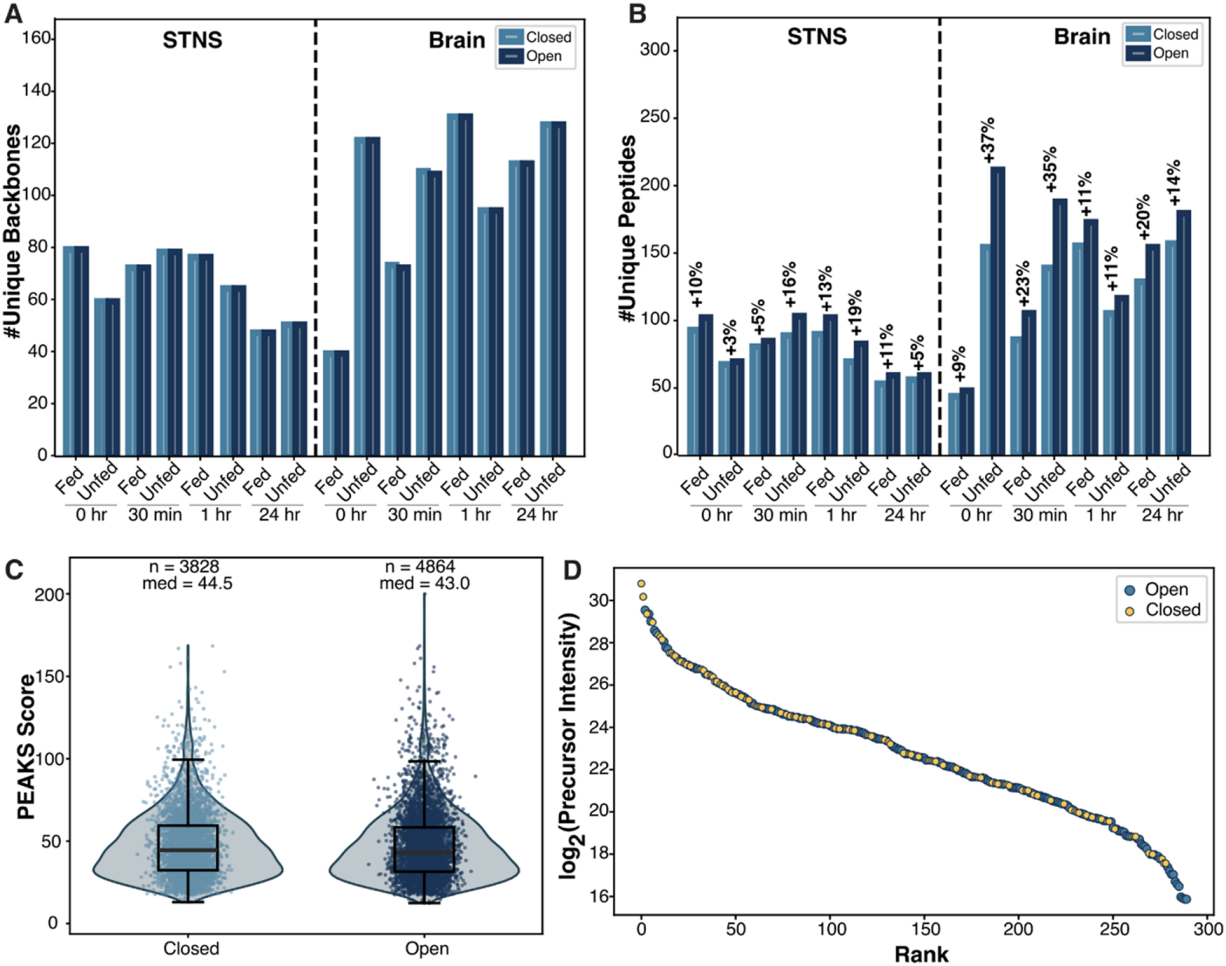
Comparison of closed and open MS^1^ search results for fed and unfed crustacean neuropeptides. (A) Unique backbones and (B) peptides identified by closed and open searches for the stomatogastric nervous system (STNS) and brain. Backbones refer to the sequence of amino acid residues stripped of any modifications, whereas peptides refers to the backbone inclusive of modifications. The times shown under each fed and unfed pair refers to the time at which the crab was sacrificed post-feeding. The percent increase in unique peptide identifications due to the open search is shown above each pair of bars. (C) Distribution of PEAKS scores (−10 log *P*) for closed and open searches. The PEAKS score refers to a score of identification confidence, with a high score indicating a more confident identification. The population statistic *n* represents the number of peptides identified by each search. (D) Dynamic range of neuropeptide intensities as identified by closed and open searches of the brain.

While the PEAKS score conveys confidence in identifications, it does not provide information about the abundance of a particular peptide. *In vivo* concentration of neuropeptides poses an ongoing challenge in analysis, where active neuropeptides can be extremely low abundance, further confounded when studying peptides with specific PTMs.^41^ Peptides from the closed and open searches were ranked according to their intensities, revealing that identifications from the open search span a wide range of intensities, seemingly filling in the gaps left by the closed search (Figures 1D, S2). This observation suggests that the open search identifies comparatively abundant neuropeptides bearing unexpected modifications that are otherwise overlooked in the closed search. Additionally, the open search also captured low-abundance peptides. Frequently, the stochastic nature of data-dependent acquisition (DDA), which selects ions for MS/MS fragmentation based on abundance, biases against detecting low-abundance neuropeptides. Thus, it is possible that the open-modification search helps mitigate this limitation with minimal additional effort by assigning spectra that would otherwise remain unannotated.^42,43^ This was further supported by MS/MS spectra showing distinct mass shifts between modified and unmodified variants of the same peptide (Figure S3). For instance, a clear mass shift corresponding to the methylation (+14.01565 Da) of an allatostatin C-type peptide was observed; this peptide was previously shown to be significantly upregulated in feeding experiments of*C. borealis*.^44^

### Improved Characterization of Modified Neuropeptides

A typical closed search can consider only a limited number of variable modifications while maintaining reasonable computation time. For neuropeptides, these commonly include C-terminal amidation, N-terminal cyclization of glutamic acid or glutamine (pyro-Glu), both of which are well-documented features of active neuropeptides.^18^ Additionally, methionine oxidation is often included in the search space, as it is a frequent sample-preparation artifact. While more specialized PTMs have been characterized only in a few cases,^45^ the well-established roles of these two terminal modifications, particularly in peptide stabilization,^46^ led us to hypothesize that they would be the most frequently observed, even in an extensive modification search. Indeed, the majority of modifications detected by the open search corresponded to at least one of these (Figure 2A). Although only a fraction of the observed modifications extended beyond our initial expectations, focusing on these revealed a diverse array of modifications occurring across a variety of amino acid residues (Figure 2B). Notably, the classification of these modifications included PTMs, chemical derivatives, nonstandard residues, and artifacts (Table S1). It is important to note that these classifications come from the UniMod database, which reports functionalities as documented in the literature.^47^ For less conventional samples, such as crustacean neuropeptides, some modifications warrant re-evaluation. For example, the aforementioned formation of pyroglutamic acid is reported as an artifactual modification, arising spontaneously during peptide isolation steps,^48^ yet this designation has been refuted in *Aplysia* neurons, where the modification occurs *in vivo* via enzymatic activity.^49^ Thus, it is important to further investigate the mechanisms underlying these modifications. Additionally, recognizing and accounting for artifactual modifications can facilitate the detection of neuropeptides that might otherwise be overlooked.

**Figure 2:**
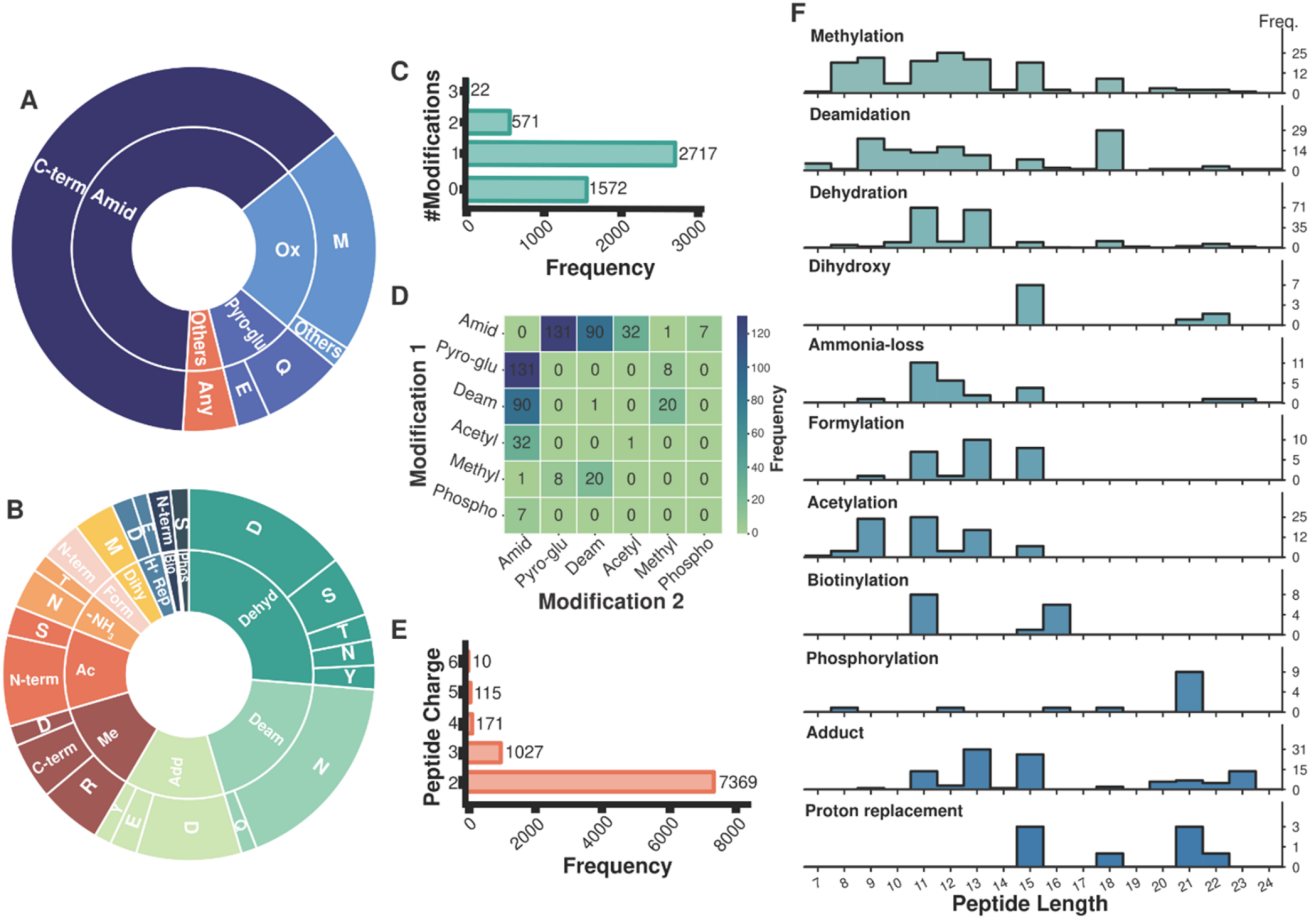
Global insight into post-translational modifications which are identified by open-modification searching. Proportional distribution of (A) all identified modifications (inner), (B) expansion of the distribution the “other” category of modifications (inner), and their site of modification (outer). Amino acid residues are abbreviated using single letter notation and modifications are abbreviated as follows: amidation (Amid), oxidation (Ox), N-termini cyclization of glutamic acid and glutamine (Pyro-glu), dehydration (Dehyd), deamidation (Deam), adduct (Add), methylation (Me), acetylation (Ac), ammonia-loss (−NH_3_), formylation (Form), dihydroxy (Dihy), proton replacement (H^+^ Rep), biotinylation (Bio), phosphorylation (Phos). (C) Frequency of the number of modifications occurring on a single peptide. (D) Heat map showing the frequency of co-occurring modifications for doubly modified peptides. Light green colors indicate low or no co-occurrence, whereas dark blue colors indicate frequent co-occurrence. The axis labels for Modifications 1 and 2 are independent of order in which the modifications were observed. (E) Frequency of charge state occurring for a peptide. (F) Frequency at which a modification occurs on a peptide of a particular length.

Among peptides identified in the open search, most contained a single modification (Figure 2C), consistent with the expected composition of biologically active neuropeptides. Moreover, many were doubly modified, accounting for more than 10% of all peptides. As “capping” modifications at both termini are common in neuropeptides, we investigated potential crosstalk between PTMs.^50,51^ As expected, the combination of N-terminal pyro-Glu and C-terminal amidation was prominent among doubly modified peptides (Figure 2D). C-terminal amidation was also frequently paired with N-terminal acetylation. Interestingly, deamidation, particularly at asparagine, was also associated with C-terminal amidation. Parsing deamidation sites can be challenging, as the mass shift (+0.984 Da), is similar to the shifts imposed by citrullination (+0.984 Da) and ^13^C isotopes (+1.0033 Da).^52,53^ Deamidation generates four Asn-derived isomeric species, a process that alters the peptide structure and is linked to significant health outcomes in mammals.^54^ Notably, several D-amino acid containing peptides (DAACPs) have also been identified in crustaceans,^55^ including crustacean hyperglycemic neuropeptides in lobsters^56^ and crayfish,^57^ with large-scale studies optimizing methodologies for in-depth DAACP profiling.^58^ Additionally, it has been reported that less-bulky C-termini are associated with an increased propensity for deamidation,^59^ which may be influenced by the frequent occurrence of C-terminal amidation that reduces steric hindrance.^46^ Given the frequent co-occurrence of deamidation and amidation, the role of deamidation in Asn isomerization, and the established presence of D-amino acids in neuropeptides, this pattern suggests potential biological crosstalk.

Supporting this hypothesis, in galanin, a short-lived neuropeptide, deamidation followed by methylation has been implicated in the formation of l-isoaspartate.^60^ The high frequency of these tandem PTMs in our data set further supports the idea that amino acid isomerization may occur within crustacean neuropeptides.

Beyond biological implications, methylation has also been reported as an artifact during neuropeptide extraction with acidified methanol, consistent with the observations in orcokinin.^61^ However, biological evidence shows that methylation of neuropeptide Y is a promising biomarker for colorectal cancer.^62^ Thus, methylation activity is broad-ranging and warrants further investigation.

Neuropeptides display wide variation in charge states and lengths, ranging from +1 up to +8 charges and from three to more than 100 residues.^18,63^ Open-modification searches revealed that most peptides were identified with a +2 charge state, with identifications decreasing as charge increased up to +6 (Figure 2E). This suggests that we are not detecting neuropeptides outside typical trends but rather expanding the existing profile. Investigation into several modifications from the open search revealed that most modifications were observed across a range of peptide lengths (Figure 2F). Methylation and deamidation displayed the greatest variability, with a slight bias toward relatively shorter peptides. The similar length distributions of peptides containing methylation and deamidation further support potential crosstalk. Other modifications, including phosphorylation, adduct formation, and proton replacement, showed slight preference for longer peptides. These findings highlight that peptides of varying lengths are dynamically modified, with no strong bias toward a particular length. Future investigations may leverage open-modification searching to probe extreme peptide lengths for these modifications.

### Connecting Observed PTMs to Biological Principles

Motifs are patterned sequences that are evolutionarily conserved due to their roles in biological processes across species, forming neuropeptide families with shared structures and functions.^64^ Because certain peptidomic motifs are associated with specific PTMs, for example, RYamides (*e.g.*, FXXXRYamide) or sulfakinins (*e.g.*, Y_(sulfo)_GHM/LRFamide), we sought to evaluate the identified peptides for conservation sequence patterns linked to the observed PTMs. Deamidation revealed four conserved regions: two upstream and two downstream of the modification site (Figure 3A). The first conserved region, AQGLGKME, begins 21 residues upstream of the deamidation site; this segment was found to correspond exclusively to CPRP peptides. Additionally, RGALEPN was identified as a putative motif in which the terminal Asn undergoes deamidation; this region also maps exclusively to the CPRP family. The most frequently observed motif among orcokinin peptides was EIDRS, located three residues downstream of the deamidation site. Finally, the fourth and fifth motifs, VMNDEA and LMNEA, both corresponded to pigment-dispersing hormones (PDH). Although deamidation of orcokinin, CPRP, and PDH peptides has been reported previously, this is the first observation of a broader conservation pattern.^64^ For methylation, NSEL (PDH), YKIFEPLR (cryptocyanin), and VMNDA (PDH) motifs were observed (Figure 3B). The conservation of the VMND/EA motif across both methylation and deamidation instances supports the previous observations of potential crosstalk between these PTMs. Dehydration also exhibited several conserved sequence regions, including NFD, EIDR, and GFGF, all of which correspond to orcokinin peptides (Figure 3C). Individually, most of the motifs observed here were previously validated as crustacean neuropeptide motifs (Table S2),^64^ though previous motif characterization did not consider the patterns in the context of modifications, focusing instead on general neuropeptide identification.

**Figure 3:**
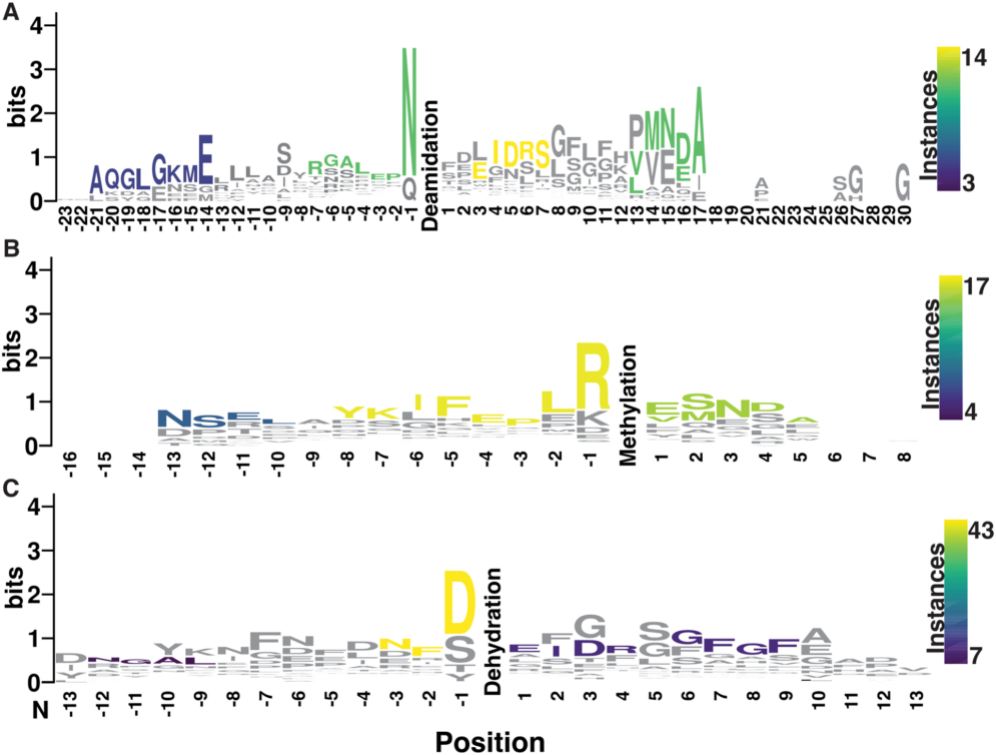
Amino acid sequence motifs for (A) deamidation, (B) methylation, and (C) dehydration. Motifs found to occur at notable frequencies were colored in a heatmap fashion, with yellow indicating a greater frequency and blue lower frequency.

It is important to recognize that PTMs may occur enzymatically or, in some cases, nonenzymatically, and amino acid motifs often help identify a peptide or peptide region as a potential substrate for modification.^65–67^ However, beyond sequence motifs, the relevant biosynthetic enzymes and their cofactors must be coexpressed at sufficient levels within the appropriate cells to enable the modification. Thus, while the motifs we identify (Figure 3) reflect patterns in which crustacean neuropeptides are modified, they are not the sole determinants of modification. Instead, they highlight potential substrates whose modification ultimately depends on the cellular context, pointing to an avenue for deeper investigation.

### Open-Modification Searching Reveals Unique Modification Patterns in Fed and Unfed Crustacean Neuropeptides Across Time

Recognizing the intricate interplay between PTMs and *in vivo* activity, we hypothesized that modifications may be differentially expressed in fundamental biological functions, such as feeding. To evaluate this, peptides with identical backbones in fed and unfed states were compared, revealing that these shared sequences diverged in their modification profiles according to feeding status (Figure 4A) across all five tissues examined: brain, STNS, TG, PO, and SG. This trend was also observed consistently over time within each tissue (Figures S4 and S5).

**Figure 4:**
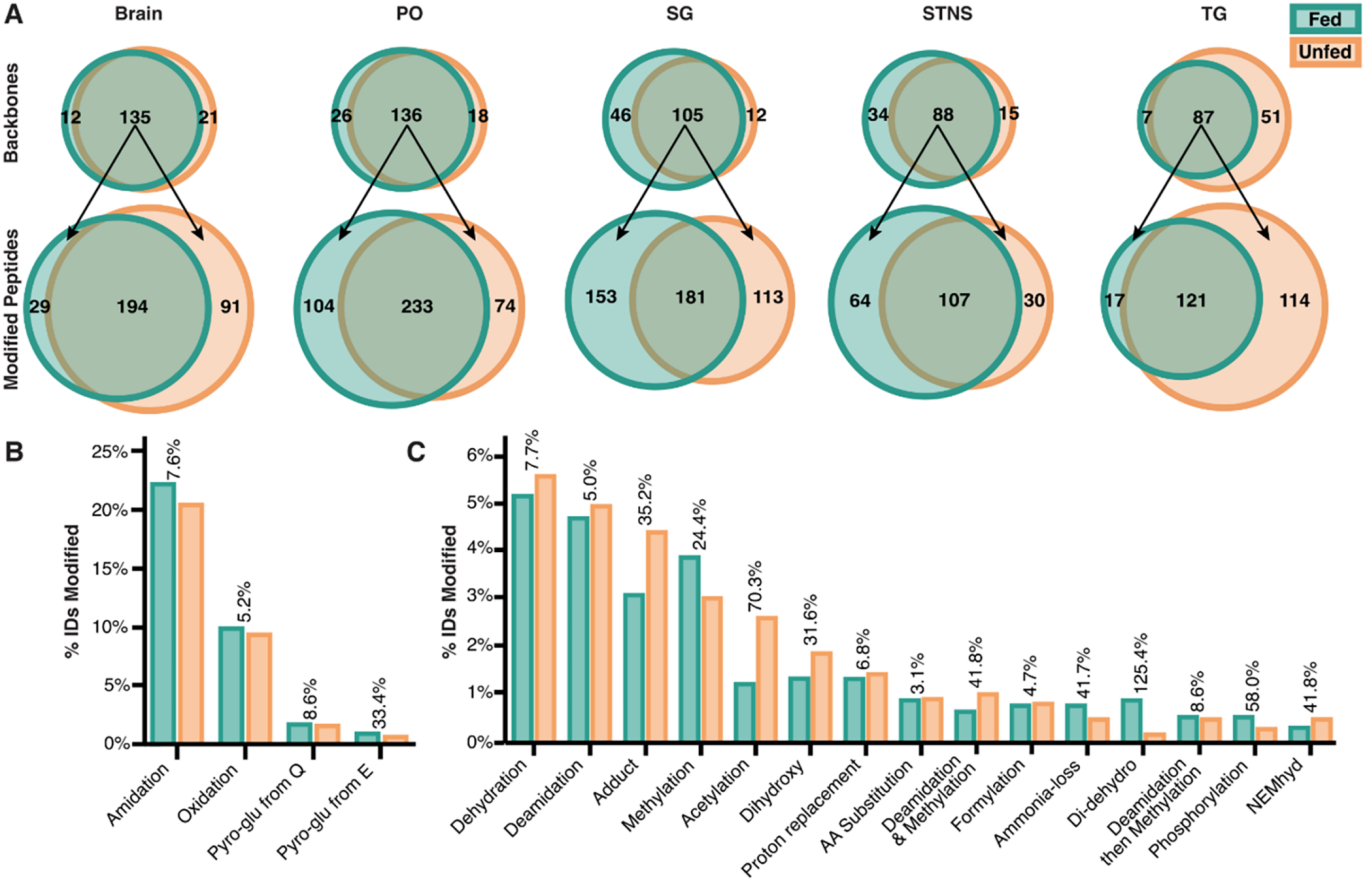
Global analysis of modified neuropeptides from fed and unfed tissues identified using open-modification searching. (A) Overlap of backbones and modified peptides for the brain, paired pericardial organs (PO), paired sinus glands (SG), stomatogastric nervous system (STNS), and thoracic ganglion (TG) across all time points. Proportion of modified peptides found with (B) common and (C) the top 15 previously uncharacterized modifications across all tissues and time points. Abbreviations: amidation (Amid), oxidation (Ox), N-termini cyclization of glutamine and glutamic acid (Pyro-glu from Q and E, respectively), amino acid (AA) substitution, didehydration (Didehydro), N-ethylmaleimidehydrolysis (NEMhyd).

A global overview of PTMs detected in fed and unfed states showed that the most abundant modifications occurred at similar frequencies in both fed and unfed states (Figure 4B). Despite this overall similarity, several less-characterized modifications exhibited more pronounced differences between states. For example, adducts (*e.g*., sodium adducts), acetylation, and dihydroxylation were more frequently detected in the unfed state, whereas didehydration was more prevalent in the fed state (Figure 4C). Some of the detected modifications are likely biological, such as acetylation^68^ and deamidation;^69^ however, other modifications, such as methylation, may arise either from biological processes^70^ or from sample preparation artifacts.^61^ Several additional modifications are almost certainly artifactual, such as sodium adducts.^31,71^ Still, the contribution of these modifications remains poorly defined, as many are sparsely characterized in the context of neuropeptides, including dehydration. Overall, these patterns suggest that certain modifications may be associated with biological activity related to feeding, while others underscore the importance of accounting for adducts -- likely originating from electrospray ionization -- to maximize neuropeptide identification.

Of the neuropeptides identified by the open search, many exhibited unique modification patterns that appeared to correspond to feeding condition (Table S3). These observations were particularly evident within several families, including allatostatin, RFamide, orcomyotropin, orcokinin, RYamide, and tachykinin neuropeptides. Notably, each of these families has been extensively documented to play biological roles within feeding-induced neuropeptide signaling cascade.^16^ From this cohort, several peptides demonstrated feeding-related modification patterns across time and feeding status, prompting further investigation (Table 1). For example, FDAFTTGFGHS, an orcomyotropin peptide, was characteristically acetylated in the brain and STNS. While N-terminal acetylation was present in the brain, acetylation at threonine, serine, cysteine, tyrosine, or histidine (TSCYH) was observed in all brain samples except at the 0 h fed time point, and it appeared exclusively in the 30 min, 1 h, and 24 h unfed STNS samples. Moreover, acetylation was consistently observed at the C-terminal serine. Acetylation of neuropeptides has been studied in mammals, where N-terminal acetylation modulates the potency of adrenocorticotrophic hormone and the activity of β-endorphins.^20,72^ Given that orcomyotropin is associated with stimulation of hindgut contraction in crayfish^73,74^ and acetylation has been linked to potency modulation, it is possible that acetylation of FDAFTTGFGHS alters the extent to which this peptide stimulates hindgut contraction. However, it is important to note that the described functions of acetylation largely refer to N-terminal modifications rather than C-terminal serine acetylation, which may operate through distinct mechanisms. Furthermore, neuropeptide acetylation beyond the N-terminus remains vastly understudied, likely due to its relatively recent discovery less than two decades ago.

**Table 1:**
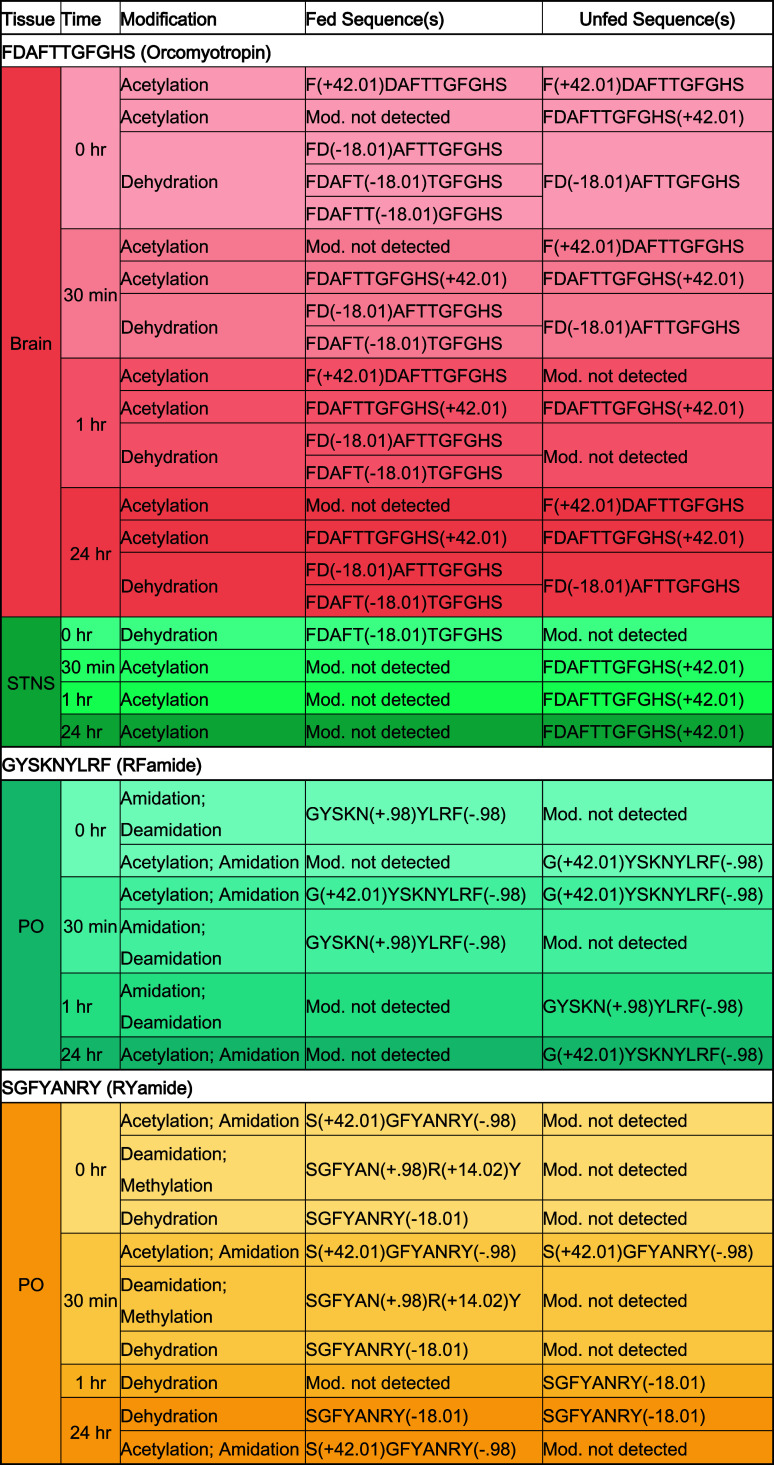
Highlighted List of Amino Acid Sequence Backbones Shared between Fed and Unfed States but Differentially Modified in a Feeding-dependent Manner

Additionally, a unique feeding-dependent pattern of deamidation and N-terminal acetylation was also observed in the RFamide peptide GYSKNYLRF in the PO across time. As expected for the conserved RFamide sequence motif, C-terminal amidation was present in all PO samples, regardless of condition. In the fed state, this peptide was deamidated at 0 and 1 h, both deamidated and acetylated at 30 min, and unmodified at 24 h. Conversely, in the unfed state, the peptide exhibited only one modification at a time: acetylation at 0 h, 30 min, and 24 h, and deamidation only at 1 h. Acetylation and deamidation are generally associated with potency modulation^20,72^ and degradation, respectively, and RFamides are known to exert anorexigenic effects across multiple organisms.^73,75,76^ These observations suggest that, in the fed state, the peptide may be deactivated at 0 h, 30 min, and 1 h to promote feeding, with altered potency at 30 min post-feeding. At 24 h when neither modification is present, the peptide may be active, contributing to appetite suppression. In the unfed state, acetylation of the peptide at 0 h, 30 min, and 24 h post-feeding, could reflect changes in potency supporting appetite suppression. These findings underscore the need for further investigation into the functional roles of these peptides with less common modifications. Finally, the RYamide peptide SGFYANRYamide demonstrated several unique modification patterns in the PO across time. This neuropeptide family is implicated in feeding through release into the hemolymph.^16^ As with RFamide, amidation was present in all PO samples regardless of time or feeding status. All fed time points except 1 h post-feeding exhibited N-terminal acetylation, whereas this modification was observed only at 30 min in the unfed PO. Based on current knowledge, N-terminal acetylation may modulate the degree to which the peptide is released into the hemolymph for downstream neural modulation of the STNS. SGFYANRY was also observed to be simultaneously deamidated and methylated exclusively at 0 h and 30 min in the fed PO. Although the functional roles of these simultaneous modifications are currently unknown, their specificity to early post-feeding time points suggests the need for deeper investigation into their potential time-dependent regulatory functions.

### Balance of Uninformed Open Searches with Biological Understanding

The application of open modification searching strategies to uncover previously unrecognized processes in complex biological matrices, such as in crustacean tissues, proved to be a valuable complement to expert-guided peptide evaluation. For example, substantial evidence demonstrates that for a biologically active neuropeptide with C-terminal amidation, the residue immediately following the cleavage site must be glycine to enable enzymatic amidation.^77^ Because this requirement is not incorporated into most search engines, current mass spectrometry workflows can yield a high false-positive rate for detecting genuinely amidated peptides.^78^ At present, however, applying this validation criterion to *C. borealis* peptides is limited by the lack of a well-annotated genome and corresponding prohormone database through which candidate neuropeptides can be contextualized. Additionally, tyrosine sulfation is a well-characterized and biologically important PTM in neuropeptidomics, with established roles in modulating GPCR recognition and activity.^18^ However, no sulfated peptides were detected in our analyses. We speculate that this absence reflects current limitations in applying proteomics-oriented software tools to neuropeptide data sets. In particular, phosphorylation (+80 Da) is far more common than sulfation (+80 Da) in proteomic workflows, and default mass-shift lists in PEAKS may not sufficiently prioritize sulfation during open-modification searches. Thus, we propose that open-modification searches represent a discovery effort, observing putative understudied modifications in neuropeptidomics (*i.e.*, deamidation, methylation), but are not a replacement for targeted experiments probing particular neuropeptides.

This work describes neuropeptides with identical amino acid sequences that exhibit a wide range of PTMs, and in some cases, no modification at all. Neuropeptides are generally considered to be highly modified molecules, with PTMs often inducing conformational changes necessary for effective binding to their cognate GPCR targets.^79^ However, there are also well-defined classes of neuropeptides whose most biologically active forms are unmodified.^80^ Taken together, these observations underscore the importance of evaluating each neuropeptide within the context of its specific modifications. We therefore propose that advancing our understanding in this area requires recognizing that differentially modified neuropeptides are likely to exhibit distinct levels of biological activity.

## Conclusions

Open-modification searching provides an efficient strategy for identifying modified peptides without the need to exponentially expand the search space. We applied this approach to crustacean neuropeptidomics and demonstrate that open searches effectively complement closed searches by assigning spectra from both abundant peptides bearing unexpected modifications and low-abundance modified species that are often missed in data-dependent acquisition workflows. By reducing the number of unassigned MS/MS spectra, this strategy improved neuropeptidome coverage and revealed modifications not previously characterized in this model system. These analytical advances revealed new biological insights, including the detection of differential PTM patterns between feeding states that were obscured when only peptide backbone sequences were considered. Notably, our results suggest potentially coordinated effects between pairs of PTMs, such as Asn deamidation and C-terminal amidation. Overall, this work establishes open-modification searching as a powerful discovery tool for neuropeptidomics, laying the foundation for targeted enrichment and functional characterization of peptides carrying underexplored modifications.

## Supplementary Material



## Data Availability

All mass spectrometry proteomics data have been deposited to the ProteomeXchange Consortium via the MassIVE partner repository with the data set identifier: MSV000099803.

## Notes

Comparison of closed and open search identifications for PO, SG, and TG (Figure S1), comparison of precursor intensities for a closed and open search in the PO, SG, STNS, and TG (Figure S2), comparison of modified and unmodified MS/MS spectra (Figure S3), overlap of backbones identified by open-modification searching for fed and unfed crustaceans over time (Figure S4), overlap of peptides identified by open-modification searching for fed and unfed crustaceans over time (Figure S5), summary of open-search-identified modifications and their Unimod classifications (Table S1), analysis of motifs associated with deamidation, methylation, and dehydration (Table S2), and comparison of fed and unfed modifications for shared backbones (Table S3) (PDF)
