# Supplementary material for: Global Profiling of Post-Translationally Modified Crustacean Neuropeptidome Trends Affiliated with Feeding Activity
Source: J Am Soc Mass Spectrom. 2026 Mar 18;37(4):997–1008. doi: 10.1021/jasms.6c00017 (PMC13047691; doi:10.1021/jasms.6c00017)
Supplement: Supplementary file 1 [file js6c00017_si_001.pdf]

Supplemental Information

Global profiling of post-translationally modified crustacean neuropeptidome trends affiliated with feeding activity

Lauren Fields,[1†] Kendra G. Selby,[1†] Meghan M. Hayes,[2] Paul Corsetti,[3] Tong Gao,[1]

Lingjun Li[1,4,5,6*]

[1]Department of Chemistry, University of Wisconsin-Madison, 1101 University Avenue, Madison, WI 53706, USA

[2]Department of Chemical and Biological Engineering, University of Wisconsin–Madison, 1415 Engineering Drive Madison, WI 53706, USA

[3]Department of Molecular & Environmental Toxicology, 1300 University Ave #6152, Madison, WI 53706, USA

[4]School of Pharmacy, University of Wisconsin-Madison, 777 Highland Avenue, Madison, WI 53705, USA

[5]Lachman Institute for Pharmaceutical Development, School of Pharmacy, University of Wisconsin-Madison, Madison, WI, 53705, USA

[6]Wisconsin Center for NanoBioSystems, School of Pharmacy, University of Wisconsin-Madison, Madison, WI 53705, USA.

[†]Equal contribution: These authors contributed equally to this work.

[*]Corresponding author

Tel.: +1 (608) 265-8491

Fax: +1 (608) 262-5345

Email: Lingjun.Li@wisc.edu

Table of Contents

Supplemental Figures (located within this document)

- **Figure S1:** Comparison of closed and open search identifications for PO, SG, and TG
- **Figure S2:** Comparison of precursor intensities for a closed and open search in the PO, SG, STNS, and TG.
- **Figure S3.** Comparison of modified and unmodified MS/MS spectra.
- **Figure S4:** Overlap of backbones identified by open-modification searching for fed and unfed crustaceans over time.
- **Figure S5:** Overlap of peptides identified by open-modification searching for fed and unfed crustaceans over time.
- **Table S1:** Summary of open-search-identified modifications and their Unimod classifications.
- **Table S2:** Analysis of motifs associated with deamidation, methylation, and dehydration.
- **Table S3:** Comparison of fed and unfed modifications for shared backbones

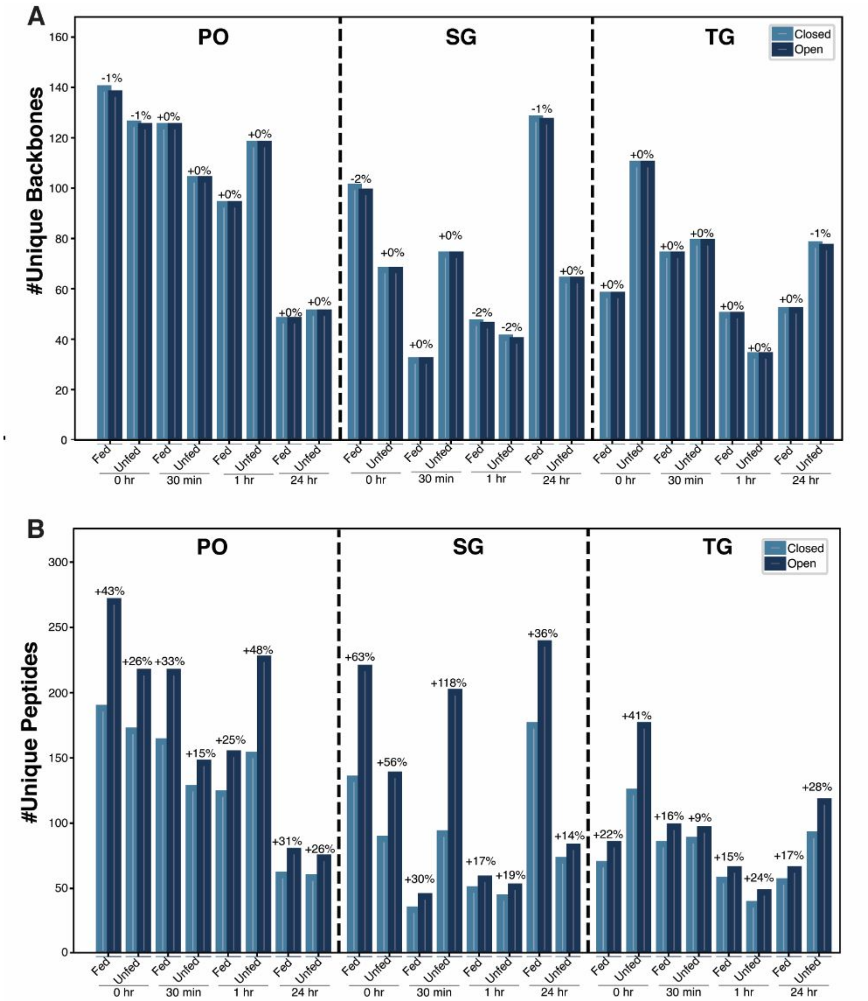

Figure S1. Comparison of unique identifications of crustacean neuropeptide backbones and peptides. **(A)** Unique backbones and **(B)** peptides identified by closed and open searches for the paired pericardial organs (PO), paired sinus glands (SG), and thoracic ganglion (TG). Note that backbones refer to the peptide sequence *sans* modifications, whereas peptides refer to the backbone and any identified modifications. The times at which the crab was sacrificed post-feeding is stated beneath the fed-unfed pairs. Shown above the fed-unfed pairs are the percent increase in (A) backbone) and (B) peptide identifications due to the open search.

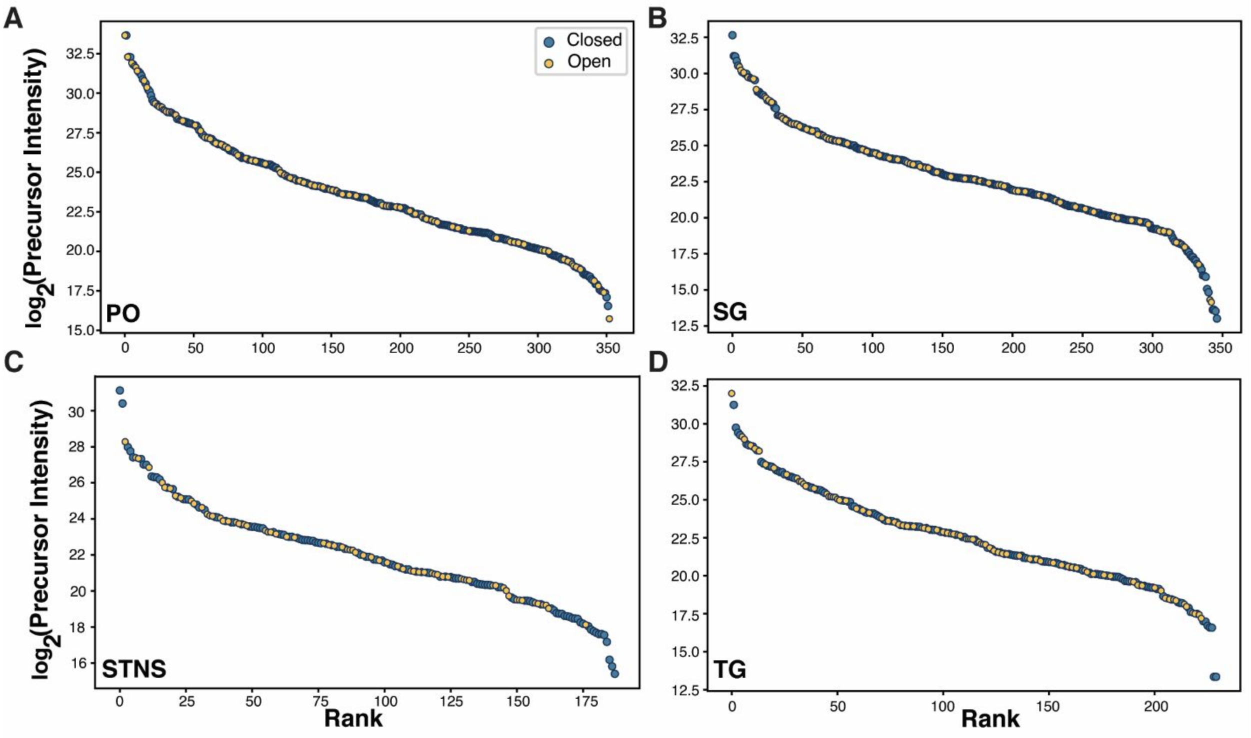

Figure S2. Comparison of neuropeptide precursor intensities due to closed and open searches. Results are shown for the paired **(A)** pericardial organs (PO), **(B)** paired sinus glands (SG), **(C)** stomatogastric nervous system (STNS) and (D) thoracic ganglion (TG).

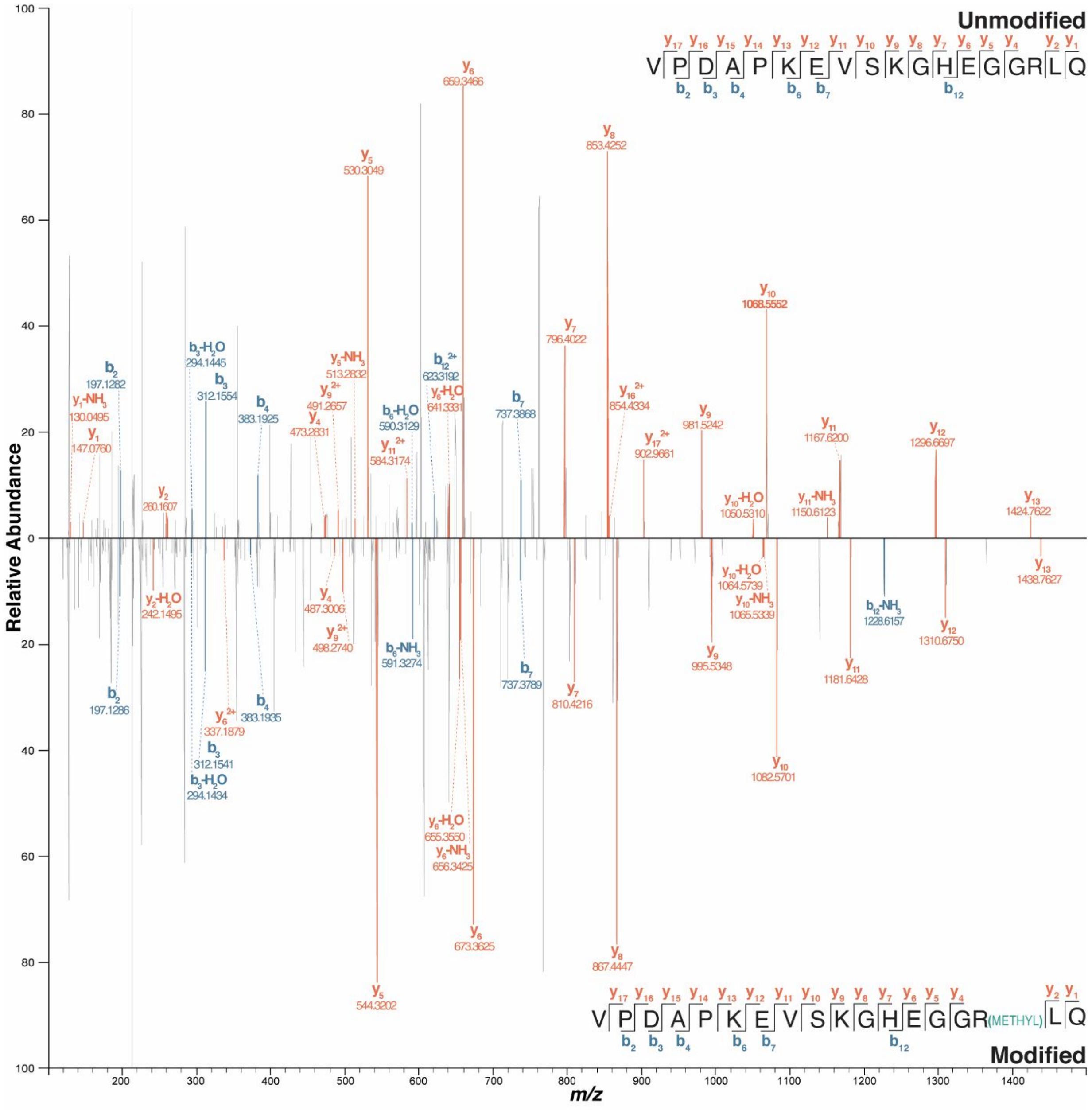

Figure S3. Comparison of modified and unmodified MS/MS spectra. Unmodified (upper) and R-methylated (+14.02; lower) spectrum for C-type Allatostatin precursor-related peptide VPDAPKEVSKGHEGGRLQ extracted from a 0 hr fed pericardial organ (PO) of *Cancer borealis*.

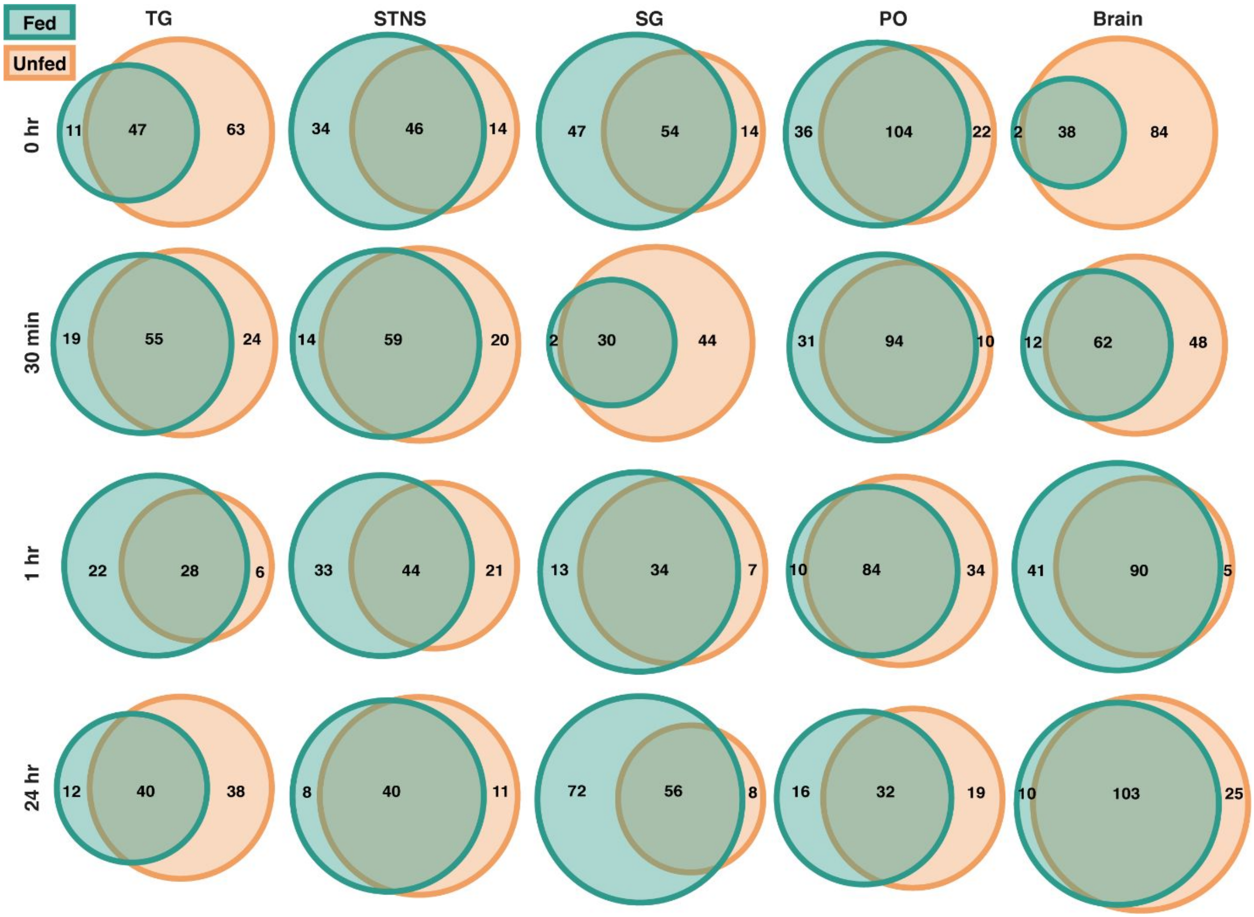

Figure S4. Overlap of backbones for the brain, paired pericardial organs (PO), paired sinus glands (SG), stomatogastric nervous system (STNS), and thoracic ganglion (TG), delineated by time points. Note that backbones refer to the peptide sequence *sans* modifications. The times shown refers to the time at which the crab was sacrificed post-feeding.

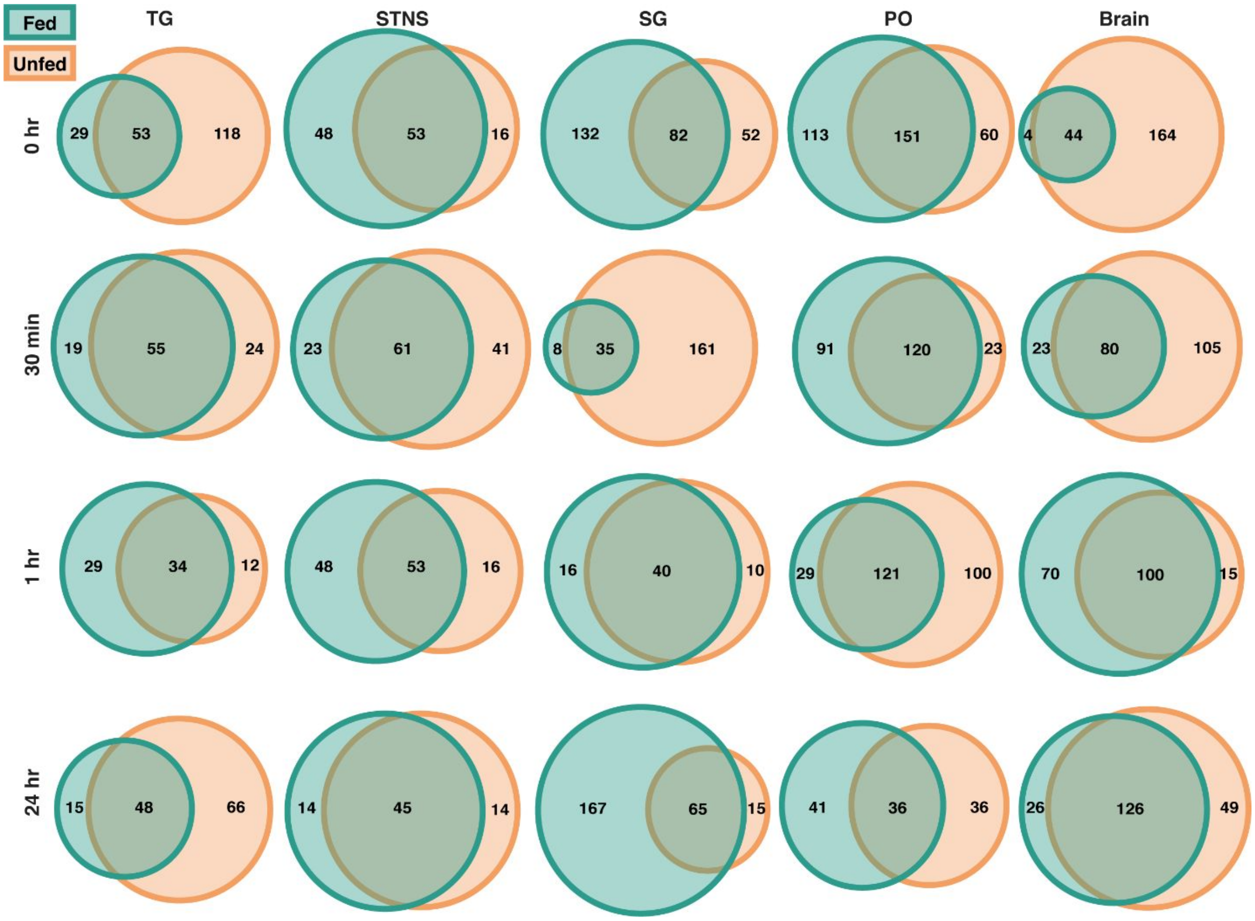

Figure S5. Overlap of peptides for the brain, paired pericardial organs (PO), paired sinus glands (SG), stomatogastric nervous system (STNS), and thoracic ganglion (TG), delineated by time points. Note that peptides refer to the backbone and any identified modifications. The times shown refers to the time at which the crab was sacrificed post-feeding.

Table S1: All modifications identified by open-modification searching and their Unimod classifications.

Modification category	Modification name	Amino Acid	Classification
AA Substitution/Modification	Alpha-amino adipic acid	K	Post-translational
	Lysine Oxidation to aminoadipic semialdehyde		
	Arginine Oxidation to glutamic semialdehyde	R	Chemical derivative
	Proline Oxidation to pyroglutamic acid	P	
	Proline Oxidation to pyrrolidinone		
	Tyrosine Oxidation to 2-aminotyrosine	Y	
	Methionine replacement by azido homoalanine	M	Non-standard residue
	Selenium replaces sulphur		
	Trifluoroleucine	L	
	Fluorination	F	
Acetylation	Acetylation (K)	K	Multiple
	Acetylation (Protein N-term)	N-terminus	Post-translational
	Acetylation (TSCYH)	S, T	
	Acetylation (Protein N-term); Amidation	N-terminus	Post-translational
	Acetylation (Protein N-term); Oxidation (M)	N-terminus	
	Acetylation (Protein N-term); Acetylation (K)	N-terminus; K	
Adduct	Levuglandinyl-lysine pyrrole Adduct	N-terminus	Post-translational
	Levuglandinyl-lysine anhyropyrrole Adduct		
	Levuglandinyl - lysine hydroxylactam Adduct		
	Levuglandinyl - arginine lactam Adduct	R	
	Levuglandinyl - arginine hydroxylactam Adduct		
	Adduct of phenylglyoxal with Arg		Chemical derivative
	MDA Adduct +54	K	
	Glycidamide Adduct	Y, M	
	Sodium Adduct	D, E, C-terminus	Artefact
Amidation	Amidation	C-terminus	

Modification category	Modification name	Amino Acid	Classification
	Acetylation (Protein N-term); Amidation		Post-translational
	Amidation; Phosphorylation (STY)		
	DeAmidation (NQ); Amidation		
	Phosphorylation (STY); Amidation		
	Pyro-glu from E; Amidation		
	Methylation(KR); Amidation		
	Pyro-glu from Q; Amidation		
	Oxidation (M); Amidation		
Ammonia-loss	Ammonia-loss (N)	N	Post-translational
Azole	Formation of five membered aromatic heterocycle	S	Post-translational
Beta-methylthiolation	Beta-methylthiolation (ND)	D	Post-translational
Biotinylation	Biotinylation	N-terminus	Chemical derivative
Carbamidomethylation	CarbamidoMethylation (DHKE X@N-term)	D, E, N-terminus	Artefact
Carbamylation	Carbamylation	K	Multiple
Carboxylation	Carboxylation (DKW)	D	Post-translational
	Carboxylation (E)	E	
Carboxymethyl	Carboxymethyl (KW X@N-term)	N-terminus	Artefact
Chlorination	Chlorination of tyrosine residues	Y	Artefact
Condensation product	Condensation product of 3-deoxyglucosone	R	Multiple
	Condensation product of glucosone		Other
Cytopiloyne	Nucleophilic addtion to cytopiloyne	P	Chemical derivative
Dansyl	5-dimethylaminonaphthalene-1-sulfonyl	R	Chemical derivative
Deamidation	DeAmidation (NQ)	N, Q	Artefact
	DeAmidation (NQ); Amidation		
	Oxidation (M); DeAmidation (NQ)		
	DeAmidation followed by esterification with ethanol		Chemical derivative
Deamidation & Methylation	DeAmidation (NQ); Methylation(KR)	Deamidation at N, Q	Artefact
	Methylation(KR); DeAmidation (NQ)	Methylation at K, R	Post-translational
Deamidation followed by a Methylation	Deamidation followed by a Methylation	Q, N	Q: Chemical derivative

Modification category	Modification name	Amino Acid	Classification
			N: Post-translational
Dehydration	Dehydration	D	Chemical derivative
		S, T, N, Y	Post-translational
Deoxy	Deoxy	S, T	Chemical derivative
Dethiomethyl	Dethiomethyl	M	Artefact
Didehydroretinylidene	Didehydroretinylidene	K	Post-translational
Didehydro	2-amino-3-oxo-butanoic_acid	T, S, Y	Post-translational
Dihydroxy	Dihydroxy	F, W	Chemical derivative
		P, Y	Post-translational
	Sulphone	M	
Ethanolamine	Carboxyl modification with ethanolamine	C-terminus	Chemical derivative
Ethanolation	Ethanolation (KR)	R	Chemical derivative
Ethylation	Ethylation	C-terminus	Chemical derivative
Formylation	Formylation	K	Artefact
	Formylation (Protein N-term)	N-terminus	Post-translational
GlycerylPE	Glycerylphosphorylethanolamine	E	Post-translational
Glycosylation	2 4-diacetamido-2 4 6-trideoxyglucopyranose	N	Post-translational
HNE	4-hydroxynonenal (HNE)	K	Post-translational
HPG (mono, bis)	Hydroxyphenylglyoxal arginine	R	Chemical derivative
	Bis(hydroxphenylglyoxal) arginine		
Iminobiotinylation	Iminobiotinylation	N-terminus	Chemical derivative
Malonylation	Malonylation	S	Chemical derivative
Menadione	Menadione quinone derivative	K	Chemical derivative
Methylation	Methylation(C-term)	C-terminus	Post-translational
	Methylation(KR)	R, K	
	Methylation(others)	D, S, E	
	DiMethylation(KR)	R	
	DiMethylation of proline residue	P	

Modification category	Modification name	Amino Acid	Classification
	Oxidation (M); Methylation(KR)	R	
	Pyro-glu from E; Methylation(KR)		
	Pyro-glu from Q; Methylation(KR)		
	Methylation(KR); Amidation		
Monoglutamyl	Monoglutamyl	E	Post-translational
NEMhyd	Nethylmaleimidehydrolysis	K	Chemical derivative
N-Homocysteine thiolactone	N-Homocysteine thiolactone	K	Post-translational
NMM	Nmethylmaleimide	K	Chemical derivative
O-Diisopropylphosphorylation	O-Diisopropylphosphorylation	K	Chemical derivative
ONE	4-Oxononenal (ONE)	K	Chemical derivative
O-Pinacolylmethylphosphonylation	O-Pinacolylmethylphosphonylation	T, Y	Chemical derivative
Oxidation	Oxidation (HW)	H	Artefact
	Oxidation (M)	M	
	Oxidation or Hydroxylation	Y, P, D, N, R	Post-translational
	Oxidation to nitro	Y	Chemical derivative
	Acetylation (Protein N-term); Oxidation (M)	M	Artefact
	Oxidation (M); DeAmidation (NQ)		
	Oxidation (M); Methylation(KR)		
	Oxidation (M); Phosphorylation (STY)		
	Phosphorylation (STY); Oxidation (M)		
	Pyro-glu from Q; Oxidation (M)		
	Oxidation (M); Amidation		
PEITC	Phenethyl isothiocyanate	M	Chemical derivative
Phosphate-ribosylation	phosphate-ribosylation	E	Post-translational
Phosphopantetheine	Phosphopantetheine	S	Post-translational
Phosphorylation	Phosphorylation (STY)	S	

Modification category	Modification name	Amino Acid	Classification
	Amidation; Phosphorylation (STY)	Y	Post-translational
	Oxidation (M); Phosphorylation (STY)		
	Phosphorylation (STY); Amidation	S	
	Phosphorylation (STY); Oxidation (M)		
Piperidination	Piperidination	S	Chemical derivative
Propionamide	Propionamide (K X@N-term)	N-term	Chemical derivative
PyMIC	3-methyl-2-pyridyl isocyanate	R	Chemical derivative
Pyridylacetyl	Pyridylacetyl	Y, K	Chemical derivative
Pyro-glu from E	Pyro-glu from E	E	Post-translational
	Pyro-glu from E; Amidation		
	Pyro-glu from E; Methylation(KR)		
Pyro-glu from Q	Pyro-glu from Q	Q	Post-translational
	Pyro-glu from Q; Methylation(KR)		
	Pyro-glu from Q; Amidation		
	Pyro-glu from Q; Oxidation (M)		
redHNE	Reduced 4-Hydroxynonenal	K	Chemical derivative
Proton replacement	Replacement of 2 protons by calcium	D, E, Y	Artefact
	Replacement of 2 protons by iron	D, E	
	Replacement of 2 protons by nickel	D, E	
	Replacement of proton by potassium	D, E	
	Replacement of proton with ammonium ion	D, E	
SMA	N-Succinimidyl-2-morpholine acetate	Y	Chemical derivative
thioacylPA	Membrane protein extraction	K	Chemical derivative
Ubiquitin	Ubiquitin	S	Other
		N-terminus	Post-translational

Table S2: Analysis of motifs identified from open search via comparison to known crustacean neuropeptide motif databases.

<table>
<tr><th>Modification</th><th>Sequence</th><th>MotifQuest[a]</th><th colspan="2">Manual Motif Database[a]</th><th>cNPDB[b]</th></tr>
<tr><td rowspan="8">Deamidation</td><td rowspan="2">AQGLGKME</td><td>RSAQGLGKMERLLVSY</td><td rowspan="2">QGLGK</td><td rowspan="2">CPRP</td><td rowspan="2">CPRP</td></tr>
<tr><td>RSAQGLGKMER</td></tr>
<tr><td>RGALEPN</td><td>N/A</td><td>EPN</td><td>CPRP</td><td>CPRP</td></tr>
<tr><td rowspan="4">EIDRS</td><td>DFDEIDRSGFG</td><td rowspan="4">EIDR</td><td rowspan="4">Orcokinin</td><td rowspan="4">Orcokinin</td></tr>
<tr><td>DFDEIDRSSFG</td></tr>
<tr><td>NFDEIDRSSF</td></tr>
<tr><td>NFDEIDRSGF</td></tr>
<tr><td>(V/L)MN(D/E)A</td><td>N/A</td><td colspan="2">N/A</td><td>PDH</td></tr>
<tr><td rowspan="5">Methylation</td><td rowspan="2">NSEL</td><td rowspan="2">SXL</td><td>NSELINAILG</td><td rowspan="2">PDH</td><td rowspan="2">PDH</td></tr>
<tr><td>NSELINSILG</td></tr>
<tr><td rowspan="2">YKIFEPLR</td><td>YKIFEPL</td><td>YKIF</td><td>Cryptocyanin</td><td rowspan="2">Cryptocyanin</td></tr>
<tr><td>KIFEPLRDKN</td><td>KIFEPL</td><td>HIGSLYRa</td></tr>
<tr><td>(E/V)(S/M)NDA</td><td>N/A</td><td colspan="2">N/A</td><td>PDH (VMNDA)</td></tr>
<tr><td>Dehydration</td><td>NGAL</td><td>N/A</td><td>GALPPS</td><td>CCHamide</td><td>others (GPSGGFNGALAR)</td></tr>
</table>

	NFD	NFDEIDRSGF	NFDEIDR	Orcokinin	Orcokinin
		NFDEIDRSSF			
	EIDRS	DFDEIDRSGFG	NFDEIDR	Orcokinin	Orcokinin
		DFDEIDRSSFG			
		NFDEIDRSSF			
		NFDEIDRSGF			
	GFGF	SSEDMPSSLGFGFN	FTTGFG	Orcomyotropin	Orcokinin
		DFDEIDRSGFG	TLTGFG	Orcomyotropin	
			FGF	Orcokinin	

[a] Similar or matched motifs identified by MotifQuest[1] and a manually curated crustacean neuropeptide motif database[2] are described.

[b] Neuropeptide families related to the motif are were identified by cross referencing crustacean neuropeptide web database tool (cNPDB).[3]

Table S3: Comprehensive list of backbones shared between fed and unfed states but differentially modified in a feeding dependent manner.

Tissue[a]	Time[a, b]	Backbone	Family[b]	Fed Modifications	Unfed Modifications
Brain	0 hr	QDDLIHLQDLED	AST B-type	Pyro-glu from Q	no modification
PO	0hr	GYSKNYLRF	RFamide	Amidation; Deamidation	Acetylation (Protein N-term); Amidation
PO	0hr	GYSKNYLRF	RFamide	no modification	
PO	0hr	MFAPLSGLPGNLRTI	AST C-type	Ammonia-loss	Deamidation

PO	0hr	MFAPLSGLPGN LRTI	AST C-type		Nucleophilic addtion to cytopiloyne
PO	0hr	MFAPLSGLPGN LRTI	AST C-type		Phenethyl isothiocyanate
PO	0hr	QRTYSFGL	AST A-type	Amidation; Pyro-glu from Q	Amidation
PO	0hr	TPDDTPEHGLQ VSED	AST B-type	Dehydration	Deamidation followed by a methylation
PO	0hr	TPDDTPEHGLQ VSED	AST B-type	Methylation (others)	
PO	0hr	VPDAPKEVSKG HEGGRLQ	AST C-type	Dehydration	Replacement of 2 protons by calcium
PO	0hr	VPDAPKEVSKG HEGGRLQ	AST C-type	Methylation (C-term)	Replacement of 2 protons by iron
PO	0hr	VPDAPKEVSKG HEGGRLQ	AST C-type	Methylation (KR)	
PO	0hr	VPDAPKEVSKG HEGGRLQ	AST C-type	Replacement of 2 protons by nickel	Sodium adduct
SG	0 hr	ALEPNTPLGDL SGSLGHPVE	CPRP	Oxidation (HW)	Carbamidomethylation
SG	0 hr	ALEPNTPLGDL SGSLGHPVE	CPRP	Oxidation or Hydroxylation	Deamidation
SG	0 hr	ALEPNTPLGDL SGSLGHPVE	CPRP		Replacement of 2 protons by iron
SG	0 hr	FDAFTTGFGHS	Orcomyotropin	Formylation	2-amino-3-oxo-butanoic_acid
SG	0 hr	FDAFTTGFGHS	Orcomyotropin	Iminobiotinylation	Acetylation
SG	0 hr	MFAPLSGLPGN LRTI	AST	no modification	Sulphone
SG	0 hr	NFDEIDRSGFG FA	Orcokinin	Deamidation	Carboxymethyl
SG	0 hr	NFDEIDRSGFG FA	Orcokinin	Methylation (C-term)	
SG	0 hr	NSELINSILGLP KVMNDA	PDH	Dehydration	Carbamylation
SG	0 hr	NSELINSILGLP KVMNDA	PDH	no modification	Deamidation (NQ); Methylation(KR)
SG	0 hr	NSELINSLLGIS RLMNEA	PDH		Deamidation (NQ)
SG	0 hr	NSELINSLLGIS RLMNEA	PDH	Replacement of proton with ammonium ion	Dehydration
SG	0 hr	RGALEPNTPLG DLSGS LGHPVE	CPRP	2-amino-3-oxo-butanoic_acid	Deamidation followed by a methylation
SG	0 hr	RGALEPNTPLG DLSGSLGHPVE	CPRP	5-dimethylamino-naphthalene	Replacement of proton

				-1-sulfonyl	with ammonium ion
SG	0 hr	RGALEPNTPLG DLSGSLGHPVE	CPRP	Adduct of phenyl-glyoxal with Arg	
SG	0 hr	RGALEPNTPLG DLSGSLGHPVE	CPRP	Amidation	
SG	0 hr	RGALEPNTPLG DLSGSLGHPVE	CPRP	Ammonia-loss	
SG	0 hr	RGALEPNTPLG DLSGSLGHPVE	CPRP	Deoxy	
SG	0 hr	RGALEPNTPLG DLSGSLGHPVE	CPRP	Dihydroxy	
SG	0 hr	RGALEPNTPLG DLSGSLGHPVE	CPRP	Proline oxidation to pyrrolidinone	
SG	0 hr	RSAQGLGKME R	CPRP	no modification	Membrane protein extraction
SG	0 hr	RSAQGLGKME RLLASY RGALEPNTPLG DLSGSLGHPVE	CPRP	2-amino-3-oxo-butanoic_acid	Amidation
SG	0 hr	RSAQGLGKME RLLASY RGALEPNTPLG DLSGSLGHPVE	CPRP	Alpha-amino adipic acid	
SG	0 hr	RSAQGLGKME RLLASYRGALE PNTPLGDLSGS LGHPVE	CPRP	Deamidation followed by a methylation	
SG	0 hr	RSAQGLGKME RLLASYRGALE PNTPLGDLSGS LGHPVE	CPRP	Dihydroxy	
SG	0 hr	RSAQGLGKME RLLASYRGALE PNTPLGDLSGS LGHPVE	CPRP	Levuglandinyl - arginine hydroxylactam adduct	
SG	0 hr	RSAQGLGKME RLLASYRGALE PNTPLGDLSGS LGHPVE	CPRP	Sulphone	
SG	0 hr	TPLGDLSGSLG HPVE	CPRP	Iminobiotinylation	Dehydration
SG	0 hr	TPLGDLSGSLG HPVE	CPRP	Sodium adduct	
SG	0 hr	YRGALEPNTPL GDLSGSLGHPV E	CPRP	3-sulfanylpropanoyl	Deamidation
SG	0 hr	YRGALEPNTPL GDLSGSLGHPV E	CPRP	Dehydration	Replacement of 2 protons by nickel

SG	0 hr	YRGALEPNTPLGDLSGSLGHPVE	CPRP	Hydroxyphenylglyoxal arginine	
SG	0 hr	YRGALEPNTPLGDLSGSLGHPVE	CPRP	Oxidation (HW)	
SG	0 hr	YRGALEPNTPLGDLSGSLGHPVE	CPRP	Pyridylacetyl	
SG	0 hr	YRGALEPNTPLGDLSGSLGHPVE	CPRP	Sodium adduct	
TG	0 hr	FDAFTTGFGHS	Orcomyotropin	Acetylation (Protein N-term)	Dehydration
TG	0 hr	FDAFTTGFGHS	Orcomyotropin	Iminobiotinylation	
TG	0 hr	YKIFEPLRESNL	Cryptocyanin	2-amino-3-oxo-butanoic_acid	Acetylation (K); Acetylation (Protein N-term)
TG	0 hr	YKIFEPLRESNL	Cryptocyanin	Deamidation; Methylation	Ammonia-loss
TG	0 hr	YKIFEPLRESNL	Cryptocyanin	N-Succinimidyl-2-morpholine acetate	Carbamidomethylation
TG	0 hr	YKIFEPLRESNL	Cryptocyanin		Dehydration
TG	0 hr	YKIFEPLRESNL	Cryptocyanin		Methylation (KR)
TG	0 hr	YKIFEPLRESNL	Cryptocyanin		O-Pinacolylmethyl-phosphonylation
TG	0 hr	YKIFEPLRESNL	Cryptocyanin		Oxidation or Hydroxylation
TG	0 hr	YKIFEPLRESNL	Cryptocyanin		Reduced 4-Hydroxynonenal
TG	0 hr	YKIFEPLRESNL	Cryptocyanin		Ubiquitin
Brain	30 min	AGWSSMRGAW	AST B-type	Amidation	Amidation; Oxidation (M)
Brain	30 min	FDAFTTGFGHS	Orcomyotropin	Iminobiotinylation	Acetylation (Protein N-term)
Brain	30 min	FDAFTTGFGHS	Orcomyotropin	O-Pinacolylmethyl-phosphonylation	Acetylation (TSCYH)
Brain	30 min	FYASLLKSDSPPQTAYLNSMFYRQD	HIGSLYR amide	no modification	Deamidation
Brain	30 min	NFDEIDRSGFGFA	Orcokinin	Ammonia-loss	Replacement of 2 protons by iron
PO	30 min	GYSKNYLRF	RFamide	Methylation (KR)	no modification
PO	30 min	MFAPLSGLPGNLRTI	AST C-type	Acetylation	Ammonia-loss

PO	30 min	MFAPLSGLPGN LRTI	AST C-type	Sodium adduct	Deamidation
PO	30 min	MFAPLSGLPGN LRTI	AST C-type		Glycidamide adduct
PO	30 min	MFAPLSGLPGN LRTI	AST C-type		Propionamide
PO	30 min	PSMYAFGL	AST A-type	Amidation	Amidation; Oxidation (M)
PO	30 min	SDEDDASDMM MMDPASYNRY V	FMRFamide-like	Phosphorylation	Sodium adduct
PO	30 min	SDEDDASDMM MMDPASYNRY V	FMRFamide-like	Replacement of 2 protons by iron	
PO	30 min	SDEDDASDMM MMDPASYNRY V	FMRFamide-like	Replacement of proton by potassium	
SG	30 min	NFDEIDRSGFG FA	Orcokinin	DiMethylation (KR)	Dehydration
SG	30 min	NFDEIDRSGFG FA	Orcokinin		Oxidation or Hydroxylation
SG	30 min	NFDEIDRSSFG FN	Orcokinin	Deamidation	Dehydration
SG	30 min	NFDEIDRSSFG FN	Orcokinin	Formylation	Oxidation or Hydroxylation
SG	30 min	NFDEIDRSSFG FN	Orcokinin		Sodium adduct
STNS	30 min	FDAFTTGFGHS	Orcomyotropin	Iminobiotinylation	Acetylation (TSCYH)
STNS	30 min	NPNMAEVLDER N	Pyrokinin	Oxidation (M)	Deamidation followed by a methylation
TG	30 min	YKIFEPLRESN	Cryptocyanin	Ammonia-loss	MDA adduct +54
TG	30 min	YKIFEPLRESN	Cryptocyanin	Dehydration	
TG	30 min	YKIFEPLRESN	Cryptocyanin	Methylation (KR)	
Brain	1 hr	APSGFLGMR	Tachykinin	Oxidation (M)	Methylation (KR)
Brain	1 hr	NFDEIDRSSF	Orcokinin	Amidation	no modification
PO	1 hr	EGFYSQRY	RYamide	Pyro-glu from E	Amidation
PO	1 hr	GYSKNYLRF	RFamide	Acetylation (Protein N-term); Amidation	Tyrosine oxidation to 2-aminotyrosine
PO	1 hr	MFAPLSGLPGN LRTI	AST C-type	Dihydroxy	2-amino-3-oxo-butanoic_acid
PO	1 hr	MFAPLSGLPGN LRTI	AST C-type	Phenethyl isothiocyanate	Sulphone

PO	1 hr	SDEDDASDMM MMDPASYNRY V	FMRFamide-like	Replacement of proton with ammonium ion	Dehydration
PO	1 hr	SDEDDASDMM MMDPASYNRY V	FMRFamide-like		Levuglandinyl - arginine hydroxylactam adduct
PO	1 hr	SDEDDASDMM MMDPASYNRY V	FMRFamide-like		Replacement of 2 protons by calcium
PO	1 hr	SDEDDASDMM MMDPASYNRY V	FMRFamide-like		Replacement of proton by potassium
PO	1 hr	SDEDDASDMM MMDPASYNRY V	FMRFamide-like		Selenium replaces sulphur
SG	1 hr	NFDEIDRSGFG FA	Orcokinin	Dehydration	Deamidation
STNS	1 hr	APSGFLGMR	Tachykinin	no modification	Acetylation (Protein N-term); Amidation
STNS	1 hr	FDAFTTGFGHS	Orcomyotropin	Sodium adduct	Acetylation
STNS	1 hr	NFDEIDRSSF	Orcokinin	Amidation	no modification
STNS	1 hr	YKIFEPLRESN	Cryptocyanin	Nethylmaleimide-hydrolysis	Ammonia-loss
STNS	1 hr	YKIFEPLRESN	Cryptocyanin		Deamidation
STNS	1 hr	YKIFEPLRESN	Cryptocyanin		Methylation (C-term)
TG	1 hr	YKIFEPLRESN	Cryptocyanin	Ammonia-loss	Lysine oxidation to aminoadipic semialdehyde
TG	1 hr	YKIFEPLRESN	Cryptocyanin	O-Pinacolylmethyl-phosphonylation	N-Succinimidyl-2-morpholine acetate
TG	1 hr	YKIFEPLRESN	Cryptocyanin	Reduced 4-Hydroxynonenal	
Brain	24 hr	HYSSLLR	HIGSLYR amide	no modification	Amidation
PO	24 hr	MFAPLSGLPGN LRTI	AST C-type	Ammonia-loss	Dihydroxy
PO	24 hr	MFAPLSGLPGN LRTI	AST C-type	Glycidamide adduct	Sodium adduct
PO	24 hr	MFAPLSGLPGN LRTI	AST C-type	Propionamide	
PO	24 hr	MFAPLSGLPGN LRTI	AST C-type	Sulphone	
PO	24 hr	SGFYANRY	RYamide	Acetylation (Protein N-term); Amidation	Tyrosine oxidation to 2-aminotyrosine

PO	24 hr	SGFYANRY	RYamide	Amidation; Phosphorylation	no modification
SG	24 hr	FDAFTTGFGHS	Orcomyotr opin	Iminobiotinylation	Sodium adduct
SG	24 hr	FDAFTTGFGHS	Orcomyotr opin	Methylation	
SG	24 hr	FDAFTTGFGHS	Orcomyotr opin	Oxidation or Hydroxylation	
SG	24 hr	GALEPNTPLGD LSGSLGHPVE	CPRP	Methylation	Replacement of 2 protons by iron
SG	24 hr	HYSSLLR	HIGSLYR amide	Amidation	no modification
SG	24 hr	NFDEIDRSGFG FA	Orcokinin	Dehydration	Formylation
SG	24 hr	NFDEIDRSGFG FA	Orcokinin	Methylation (C-term)	
SG	24 hr	NFDEIDRSGFG FA	Orcokinin	Oxidation or Hydroxylation	
SG	24 hr	NFDEIDRSGFG FV	Orcokinin	Dehydration	Formylation

[a] No shared backbones with unique differential modifications were identified for the 0 hr STNS, 24 STNS, or 0 hr TG.

[b] Abbreviations: pericardial organ (PO, sinus gland (SG), stomatogastric nervous system (STNS), thoracic ganglion (TG), allatostatin (AST) crustacean hyperglycemic hormone (CHH)

References

(1) Dang, T. C.; Fields, L.; Li, L. MotifQuest: An Automated Pipeline for Motif Database Creation to Improve Peptidomics Database Searching Programs. *J Am Soc Mass Spectrom* **2024**, 35 (*8*), 1902-1912. DOI: 10.1021/jasms.4c00192.

(2) Fields, L.; Vu, N. Q.; Dang, T. C.; Yen, H. C.; Ma, M.; Wu, W.; Gray, M.; Li, L. EndoGenius: Optimized Neuropeptide Identification from Mass Spectrometry Datasets. *J Proteome Res* **2024**, 23 (*8*), 3041-3051. DOI: 10.1021/acs.jproteome.3c00758.

(3) Tran, V. N. H.; Duong, T. U.; Fields, L.; Tourlouskis, K.; Beaver, M.; Li, L. cNPDB: A comprehensive empirical crustacean neuropeptide database. *bioRxiv* **2025**. DOI: 10.1101/2025.07.29.667494.
